# SUMO4 Gene SNP rs237025 and the Synergistic Effect With Weight Management: A Study of Risk Factors and Interventions for MetS

**DOI:** 10.3389/fgene.2021.786393

**Published:** 2021-12-10

**Authors:** Zhoujie Tong, Jia Qi, Weixuan Ma, Di Wang, Boang Hu, Yulin Li, Xu Jia, Jie Peng, Zhihao Wang, Ming Zhong

**Affiliations:** ^1^ The Key Laboratory of Cardiovascular Remodeling and Function Research, Chinese Ministry of Education, Chinese National Health Commission and Chinese Academy of Medical Sciences, The State and Shandong Province Joint Key Laboratory of Translational Cardiovascular Medicine, Department of Cardiology, Qilu Hospital, Cheeloo College of Medicine, Shandong University, Jinan, China; ^2^ Department of Cardiology, Zibo Central Hospital, Zibo, China; ^3^ Key Laboratory of Cardiovascular Proteomics of Shandong Province, Department of Geriatric Medicine, Qilu Hospital of Shandong University, Jinan, China

**Keywords:** MetS, SUMO4, SNP, weight management, diabetes

## Abstract

**Aim:** Metabolic Syndrome (MetS) is widespread across the world. Gene targeted therapy and risk management are promising approaches for MetS intervention. *SUMO4* gene rs237025 polymorphism is related to an increased risk of diabetes, therefore, it is considered a target for the gene polymorphism research of MetS.

**Methods:** A case-control study was performed to study the interaction of rs237025 with MetS and the components of MetS. A 5-years follow-up survey was carried out to elucidate the crosstalk between rs237025 and weight management, and the synergistic effect on MetS.

**Results:** A total of 1,008 MetS patients and 1,047 controls were recruited in this research. By cross-section study, we find that rs237025 is an independent risk factor for increased Waist Circumference (WC), elevated Triglyceride (TG), elevated Fasting Plasma Glucose (FPG), and MetS. Cross-over analysis identifies the interaction of rs237025 and weight management as a risk factor for MetS, the synergistic effects of rs237025 and weight management are negative to WC, TG, and High-density Lipoprotein-cholesterol (HDL-c).

**Conclusion:** *SUMO4* gene rs237025 is related to increased risk of MetS, weight management is essential to MetS intervention, especially for patients with rs237025 polymorphism.

## Introduction

A set of metabolic disorders characterized by glucose intolerance and lipid dysregulation was defined as MetS, which is the most prevalent non-contagious disease in the Western world nowadays (Eckel et al., 2005). The economy of China has thrived for decades, unhealthy diets and sedentary lifestyles have been widely accepted, and the abdominal obesity rate reached 29.1% (Zhang et al., 2019). According to China’s seventh national census, the population aged 60 and above accounted for 18.7%. The onset of MetS is closely related to obesity, unhealthy lifestyle, and aging (Kuk and Ardern, 2010; Lao et al., 2014), consequently, it has been reported that 24.5% of the population in China is suffering from MetS (Li et al., 2016). MetS patients are at high risk of Type 2 Diabetes (T2D), and the incidence of cardiovascular disease increases by 2-times, and the overall mortality of MetS patients is 1.5-times that of the general population (Mottillo et al., 2010). Recent studies have also related MetS with an increased risk of malignancy (Bishehsari et al., 2020). Therefore, risk prediction and post-diagnose management of MetS are of high significance.

MetS is a multifactorial diagnosis in which dyslipidemia and glucose dysregulation play a central role (Grundy et al., 2005). Glucose intolerance and disposition of adipose tissue are mutually promoted and coordinately contributory to the pathogenesis of MetS (Johnson and Olefsky, 2013). Therefore, molecular mechanisms related to dysregulated glucose metabolism are potential targets for MetS study. We have noticed that cytoplasmic SUMO4 protein combines and modifies IκBα, a transcription factor that specifically inhibits NF-κB-dependent transcription of IL-12B, which is known as a stimulatory factor of natural killer cells (Guo et al., 2004). M55V is the substitution of methionine for valine in the 55th amino acid of SUMO4. M55V substitution results in a 5.5-fold increase in NF-κB transcription, while the expression of IL-12B is trebled, which consequently lead to an autoimmune response and Type 1 Diabetes (T1D) (Guo et al., 2004). Studies have also related M55V substitution to an increased prevalence of T2D (Sozen et al., 2014; Li et al., 2017). Glucose metabolism disorder and insulin resistance play an important role in MetS, and we anticipate that study on the M55V substitution will provide new insight into MetS.

Single Nucleotide Polymorphism (SNP) research is still the essential tool for widespread mutations. *SUMO4* gene located on chromosome6 NC_000006.12. SNP rs237025 is a functional nonsynonymous substitution of 163A > G in the CUE domain of *SUMO4* which leads to M55V. This study is looking into the effects of rs237025 polymorphism on MetS, and to explore the interaction of lifestyle intervention and gene polymorphism and the synergistic effect on MetS. This study may provide new evidence to risk assessment and gene-targeted therapy for MetS.

In general, this study concentrated on the single nucleotide polymorphism of *SUMO4* gene rs237025 locus (M55V), to elucidate the interaction of rs237025 and MetS in a cross-section study. And a follow-up survey was conducted to explore the gene-environment interaction through cross-over analysis.

## Materials and Methods

### Participant Recruitment and Information Collection

This is a prospective case-control study, sample size of this work was determined based on the previously reported 15.1% estimated prevalence of MetS, 24.8% abdominal obesity rate (Gu et al., 2005), and 30.0% frequency of the G allele in China population (Lin et al., 2007). A minimum sample size of 914 case-control pairs was generated by Quanto software (University of Southern California). Participants were Shandong China Han population recruited in 2007, randomly a rural area (Xinzhai Village, Diao Town) and an urban area (Zhangqiu District, Jinan City) in Shandong province were selected. The village and the urban community each containing 1,000–1,500 households were investigated. Investigators went door-to-door for data collection. Apart from families unwilling to participant in this research, one adult subject was randomly selected in each household. Firstly, basic information, pharmaceutical history, and living habits were collected by questionnaire. Physical measurements including weight, height, WC, and resting blood pressure were performed three times by qualified investigators. Fasting blood was obtained in the morning and immediately analyzed in a standard laboratory, testing items include TG, Total Cholesterol (TC), HDL-c, and Low-density Lipoprotein-cholesterol (LDL-c). FPG was measured by Beckman Coulter LX20 chemical analyzer (Beckman Coulter, Brea, CA). Diagnose of MetS refers to the jointly establish criteria of IDF and AHA/NHLBI in 2009: ① Abdominal obesity for East Asian: WC ≥ 90 cm for men and WC ≥ 80 cm women; ② Blood TG ≥ 1.70 mmol/L or receiving TG-lowering treatment; ③ Blood HDL-c < 1.0 mmol/L for male, blood HDL-c < 1.3 mmol/L for female or receiving statin treatment; ④ SBP≥130 mmHg, or DBP≥85 mmHg, or currently receiving antihypertensives; ⑤ FPG ≥ 5.6 mmol/L, or receiving antidiabetics. Metabolic syndrome can be diagnosed if three or more components are determinate. Participants who met the diagnosis criteria were assigned into the MetS group, otherwise into the control group, for each MetS patient recruited, a control subject of similar sex and age was recruited. Exclusion criteria of this research include: confirmed malignant disease, heart failure, kidney failure, chronic hepatitis, cerebrovascular disease, severe valvular heart disease, hypertension caused by endocrine abnormalities, obstructive sleep apnea-hypopnea syndrome, and vascular stenosis, and participants with missing key information were also excluded. Genomic DNA was extracted and purified by DNA extraction kit D3133-03 (Magen, Guangzhou, China), and rs237025 polymorphism was genotyped by Sequenom MassArray genotyping system (Sequenom, San Diego, CA).

Participants with anamnesis or medical conditions diagnosed in the cross-section survey were advised to have lifestyle intervention and specialist consultation. Salt restriction, smoking cessation, regular exercise, taking diet, and psychological consulting were recommended for lifestyle intervention. Statins such as Rosuvastatin, Atorvastatin, Fluvastatin, Simvastatin were recommended for dyslipidemia. Antihypertensives such as Metoprolol, Nifedipine, Amlodipine, Captopril, Valsartan, Telmisartan, Hydrochlorothiazide, Indapamide, and Reserpine were used for hypertention. Metformin, Gliclazide, Glitaqualone, Glipizide, Pioglitazone, Acarbose, and Insulin were used as antidiabetics. A 5-years follow-up survey was performed in 2012. Physical information and fasting blood were obtained in the 5-years follow-up survey, the questionnaire format and laboratory examination were consistent with the cross-sectional study. Information regarding changes in lifestyle and pharmaceutical treatment was also collected. Weight management was defined as ΔWeight<0 kg for the 5-years follow-up survey.

### Statistics

All statistical analysis was performed using SPSS 26.0 (Chicago, Illinois). Two-tailed *p* values less than 0.05 were considered statistically significant. Hardy-Weinberg equilibrium of SNP rs237025 in this research was tested by χ^2^ goodness of fit test. Continuous variants that follow the normal distribution were presented as $\bar{\text{x}}\pm \text{S}$ , and tested by independent-sample *t* test. Categorical variables were presented as percentages and tested by χ^2^ goodness of fit test. Counting variables were presented as median [25%, 75%] [M (Q_1_, Q_3_)] and analyzed by non-parametric test. Risk factors of MetS and MetS components were analyzed by multivariate logistic regression. Changes in MetS related parameters in the 5-years survey were calculated and presented as “Δ”. The statistical power of the baseline changes in the follow-up survey was calculated by Power and Sample Size Program (William D. Dupont and Walton D. Plummer). MetS component which was “newly diagnosed” was redefined as 2, “maintaining” was redefined as 1, and “relieving” was redefined as 0, by which MetS components were reassigned and analyzed in general linear regression. Afterward, cross-over analysis was performed to analyze the gene-environment interaction. A synergistic index over one was considered a positive interaction between gene and environment, and a synergistic index less than one referred to a negative interaction between gene and environment.

## Results

### Cross-Section Analysis

This study recruited 1,008 MetS patients and 1,047 controls in the cross-section analysis, the MetS group and the control group were matched in sex and age (Table 1), and distributions of M55V (*SUMO4*, SNP rs237025, A > G) genotypes in MetS group (χ^2^ = 5.026, *p* = 0.081), control group (χ^2^ = 0.504, *p* = 0.777) and the total crew (χ^2^ = 4.637, *p* = 0.098) follow the Hardy-Weinberg equilibrium (Supplementary Table S1). The physical parameters and experimental indicators were compared thereafter, height, weight, WC, Body Mass Index (BMI), Systolic Blood Pressure (SBP), Diastolic Blood Pressure (DBP), TG, LDL-c, and FPG were significantly increased in the MetS group, while the HDL-c was significantly decreased (*p* < 0.05; Table 1). No difference was found in alcohol consumption, but the percentage of smokers was significantly higher in the control group than the MetS group (*p* < 0.001; Table 1).

**TABLE 1:** Baseline information of the control group and the MetS group.

	Control (n = 1,047)	MetS (n = 1,008)	*p*
Age (years)	51.24 ± 10.01	51.73 ± 9.61	0.261
Sex (male%)	46.32%	44.05%	0.300
Drink %	19.01%	21.23%	0.209
Smoke %	21.01%	13.79%	<0.001
Height (cm)	161.91 ± 7.67	162.71 ± 7.77	0.019
Weight (kg)	58.63 ± 8.68	72.51 ± 10.95	<0.001
WC (cm)	76.29 ± 6.72	91.19 ± 8.44	<0.001
BMI (kg/m^2^)	22.34 ± 2.76	27.35 ± 3.42	<0.001
SBP (mmHg)	119.90 ± 10.06	149.49 ± 19.74	<0.001
DBP (mmHg)	75.19 ± 6.94	90.86 ± 10.76	<0.001
TC (mmol/L)	4.16 ± 0.58	4.71 ± 1.19	<0.001
TG (mmol/L)	0.97 ± 0.46	2.36 ± 1.65	<0.001
HDL-c (mmol/L)	1.58 ± 0.34	1.49 ± 0.53	<0.001
LDL-c (mmol/L)	2.58 ± 0.54	3.21 ± 0.83	<0.001
FPG (mmol/L)	4.63 ± 0.64	6.00 ± 2.13	<0.001

MetS, metabolic syndrome; BMI, body mass index; WC, waist circumference; SBP, systolic blood pressure; DBP, diastolic blood pressure; TG, triglyceride; TC, total cholesterol; HDL-c, High-density lipoprotein-cholesterol; LDL-c, low-density lipoprotein-cholesterol; FPG, fasting plasma glucose.

The baseline characteristics of rs237025 genotypes were studied. Under the dominant model, SBP, TC, LDL-c, and FPG are significantly elevated in the AG/GG genotype group; under the recessive model, WC, LDL-c, and FPG are significantly elevated in the rs237025 GG genotypes. Subgroup studies were performed in the MetS group and the control group. Under the dominant model, SBP, and TG are significantly elevated in AG/GG genotypes in the control group; no difference was found in the MetS group. Under the recessive model, TG is significantly elevated in GG genotypes in the control group (*p* = 0.037; Supplementary Table S2); in the MetS group, WC (*p* = 0.036; Supplementary Table S2), and FPG (*p* = 0.017; Supplementary Table S2) are increased in GG genotypes compared to AA/AG genotypes.

Furthermore, χ^2^ test was performed to compare the prevalence of MetS among rs237025 genotypes, there is no significant difference in the frequency of MetS under the dominant model and the co-dominant model, while under the recessive model, the frequency of MetS is significantly increased in GG genotypes (χ^2^ = 4.743; *p* = 0.029; Table 2). Accordingly, rs237025 as an independent variable is included in the logistics regression equation under the recessive model [OR 1.470 (95% CI 1.010–2.140); *p* = 0.044; Table 3], rs237025 as an independent variable is excluded under the dominant model or co-dominant model.

**TABLE 2:** Distribution of M55V genotypes under a recessive model.

	AA/AG		GG		χ^2^	*p*
	Counts	Frequency (%)	Counts	Frequency (%)		
Control	954	91.12	93	8.88	4.743	0.029
MetS	889	88.19	119	11.81		
Normal WC	1,037	91.29	99	8.71	7.042	0.008
Increased WC	806	87.70	113	12.30		
Normal BP	831	90.42	88	9.58	0.986	0.321
Elevated BP	1,012	89.08	124	10.92		
Normal TG	1,213	90.79	123	9.21	5.082	0.024
Elevated TG	630	87.62	89	12.38		
Normal HDL	1,518	89.72	174	10.28	0.011	0.916
Decreased HDL	325	89.53	38	10.47		
Normal FPG	1,394	90.87	140	9.13	9.258	0.002
Elevated FPG	449	86.18	72	13.82		

MetS, metabolic syndrome; WC, waist circumference; BP, blood pressure; TG, triglyceride; HDL-c, high-density lipoprotein-cholesterol; FPG, fasting plasma glucose.

**TABLE 3:** Logistic regression analysis of MetS and components of MetS.

		*p*	OR	OR 95% C.I.	
				Lower limit	Upper limit
MetS					
	rs237025	0.044	1.470	1.010	2.140
	Weight (kg)	<0.001	1.156	1.140	1.172
	TC (mmol/L)	<0.001	1.888	1.659	2.149
Increased WC					
	rs237025	0.018	1.753	1.103	2.786
	BMI (kg/m^2^)	<0.001	1.669	1.575	1.770
	SBP (mmHg)	<0.001	1.039	1.028	1.051
	DBP (mmHg)	<0.001	1.073	1.053	1.094
	TG (mmol/L)	<0.001	1.229	1.096	1.378
Elevated TG					
	rs237025	0.049	1.399	1.002	1.952
	Weight (kg)	<0.001	1.102	1.090	1.113
	HDL-c (mmol/L)	<0.001	2.191	1.736	2.766
Elevated FPG					
	rs237025	0.046	1.414	1.005	1.988
	WC (cm)	<0.001	1.053	1.040	1.066
	SBP (mmHg)	<0.001	1.020	1.014	1.026
	TG (mmol/L)	<0.001	1.305	1.202	1.417

MetS, metabolic syndrome; rs237025, AA/AG = 0, GG = 1; TC, total cholesterol; WC, waist circumference; BMI, body mass index; SBP, systolic blood pressure; DBP, diastolic blood pressure; TG, triglyceride; HDL-c, high-density lipoprotein-cholesterol; FPG, fasting plasma glucose.

Thereafter, we studied the interaction of rs237025 with the components of MetS under recessive model. The distribution of AA/AG and GG is significantly different in the normal WC group/increased WC group (χ^2^ = 7.042; *p* = 0.008; Table 2), normal TG group/increased TG group (χ^2^ = 5.082; *p* = 0.024; Table 2), and in normal FPG group/increased FPG group (χ^2^ = 9.258; *p* = 0.002; Table 2). Correspondingly, logistic regression analysis shows that rs237025 is an independent risk factor for increased WC [OR 1.753 (95% CI 1.103–2.786); *p* = 0.018; Table 3], increased TG [OR 1.399 (95% CI 1.002–1.952); *p* = 0.049; Table 3], and increased FPG [OR 1.414 (95% CI 1.005–1.988); *p* = 0.046; Table 3]; non-parametric tests indicate that the number of MetS risk factors is significantly increased in GG genotypes than in AA/AG genotypes [GG 3 (0, 3), AA/AG 2 (0, 3); *p* = 0.010].

### Follow up Study

There were 269 controls and 871 MetS patients who participated in the 5-years follow-up survey, and 26 confirmed deaths. Among these 1,166 participants (deceased included), 22 were diagnosed with cerebrovascular disease, 13 with coronary artery disease, 2 with heart failure, and 20 with tumor. The distribution pattern of rs237025 genotypes in the recessive model was not significantly different in death (χ^2^ = 0.013; *Fisher* = 1.000), cerebrovascular disease (χ^2^ = 0.730; *Fisher* = 0.334), coronary artery disease (χ^2^ = 3.142; *Fisher* = 0.064), heart failure (χ^2^ = 0.524; *Fisher* = 1.000), and tumor (χ^2^ = 0.998; *Fisher* = 0.498). Among 1,140 participants in the follow-up survey (deceased excluded), the statistical power of ΔWC, ΔSBP, ΔDBP, ΔTG, and ΔHDL-c are over 0.800, and the statistical power of ΔFPG is 0.074. Participants with health issues were advised to make lifestyle changes and have pharmaceutical therapy. Consequently, weight, WC, SBP, DBP, TG, TC, LDL-c, and FPG are significantly decreased in the MetS group than in the control group. Intriguingly, HDL-c is reduced significantly in the MetS group (*p* = 0.019; Table 4), but rs237025 is excluded from the linear regression equation with ΔHDL-c as the dependent variable.

**TABLE 4:** Changes of the MetS related indicators in the follow-up survey.

	Control (n = 269)	MetS (n = 871)	*p*
ΔWeight (kg)	−0.32 ± 3.28	−2.04 ± 3.01	<0.001
ΔWC (cm)	−0.84 ± 3.73	−4.24 ± 3.26	<0.001
ΔSBP (mmHg)	1.45 ± 7.58	−7.41 ± 13.18	<0.001
ΔDBP (mmHg)	−0.05 ± 3.22	−5.62 ± 6.81	<0.001
ΔTC (mmol/L)	0.02 ± 1.06	−0.32 ± 1.38	<0.001
ΔTG (mmol/L)	0.11 ± 0.68	−0.80 ± 1.84	<0.001
ΔHDL-c (mmol/L)	−0.20 ± 0.46	−0.30 ± 0.67	0.019
ΔLDL-C (mmol/L)	−0.07 ± 0.93	−1.00 ± 0.98	<0.001
ΔFPG (mmol/L)	0.37 ± 1.53	−0.02 ± 2.57	0.021

MetS, metabolic syndrome; WC, waist circumference; SBP, systolic blood pressure; DBP, diastolic blood pressure; TG, triglyceride; TC, total cholesterol; HDL-c, high-density lipoprotein-cholesterol; LDL-c, low-density lipoprotein-cholesterol; FPG, fasting plasma glucose.

There is no difference in the number of newly diagnosed MetS in GG genotypes and AA/AG genotypes (χ^2^ = 2.808, *p* = 0.094), and changes in the number of MetS components do not show any difference (*p* = 0.959). Since MetS related parameters are highly affected by drug administration, crosstalk between drug and rs237025 on MetS was studied thereafter. There were 564 participants who received antihypertensives, 150 participants who received antidiabetics, and 63 who received statins. χ^2^ analysis indicates that the distribution of rs237025 genotypes and participants who received antihypertensives, antidiabetics, and statins, did not show statistical differences (*p* > 0.05). Factorial analysis indicates that there is no significant interaction of rs237025 and antihypertensives on ΔSBP (F = 2.114, *p* = 0.146) and ΔDBP (F = 0.333, *p* = 0.564), there is no significant interaction of rs237025 and antidiabetics on ΔFPG (F = 0.014, *p* = 0.906), and there is no significant interaction of rs237025 and statins on ΔLDL-c (F = 0.002, *p* = 0.965). χ^2^ analysis has found that in participants without drug administration, the number of new cases with decreased HDL-c are significantly increased in AA genotypes (χ^2^ = 5.154; *p* = 0.023; Supplementary Table S3). Accordingly, logistic regression has identified rs237025 as an independent risk factor for decreased HDL-c [OR 2.058 (95% CI 1.143–3.707); *p* = 0.016; Supplementary Table S4].

MetS is a multifactorial disease, which is related to heredity and largely affected by the environment and drug administration. We have considered bodyweight as a cumulative indicator of time and environment. In the subsample of weight management group (n = 1,000) and no weight management group (n = 140), the statistical power of ΔWC, ΔSBP, ΔDBP, ΔTG, and ΔHDL-c are over 0.800, and the statistical power of ΔFPG is 0.064. Logistic regression indicated that salt restriction, diet, exercise, antidiabetics, and antihypertensives are protective factors for weight management (*p* < 0.05; Supplementary Table S5). In turn, we studied the concomitant effects of drug administration on MetS related parameters by covariance analysis. The cross-talk of rs237025 and weight management is related to FPG (*p* = 0.044; Table 5), however, the statistical power of ΔFPG is 0.064, which may invalid the result.

**TABLE 5:** Covariance analysis of MetS components in the 5-years follow-up survey.

	WM		No WM		*p*							
	GG (n = 171)	AA/AG (n = 829)	GG (n = 23)	AA/AG (n = 117)	Sex	Age	AH	AD	Statin	M55V	WM	M55V*WM
WC	77.54 ± 7.34	89.93 ± 9.13	78.58 ± 5.61	90.84 ± 9.11	<0.001	0.306	<0.001	0.050	0.685	0.086	<0.001	0.075
SBP	129.25 ± 21.31	145.20 ± 21.79	128.46 ± 20.08	144.72 ± 22.17	0.298	<0.001	<0.001	0.726	0.569	0.136	0.570	0.518
DBP	78.73 ± 10.07	89.13 ± 11.45	78.54 ± 10.52	88.58 ± 10.80	0.062	0.022	<0.001	0.023	0.164	0.793	0.039	0.874
TG	1.55 ± 1.55	2.18 ± 1.60	1.21 ± 0.77	2.27 ± 1.73	0.010	0.223	0.001	0.733	0.752	0.899	0.046	0.882
HDL-c	1.56 ± 0.44	1.50 ± 0.51	1.47 ± 0.58	1.51 ± 0.47	0.720	<0.001	0.336	0.026	0.014	0.694	0.542	0.860
FPG	5.47 ± 2.13	5.66 ± 1.85	4.87 ± 0.99	6.12 ± 2.27	<0.001	0.014	0.007	<0.001	0.652	0.673	0.301	0.044

WM, weight loss = 0, weight loss = 1; AH, no antihypertensive = 0, antihypertensive = 1; AD, no antidiabetic = 0, antidiabetic = 1; Statin, no statin = 0, statin = 1; WC, waist circumference; SBP, systolic blood pressure; DBP, diastolic blood pressure; TG, triglyceride; HDL-c, high-density lipoprotein-cholesterol; FPG, fasting plasma glucose.

Thereafter, we studied changes in MetS and MetS components in liner regression model, and considered the product of rs237025 and weight management as an independent variant. The rs237025*WM is an independent risk factor for new-onset MetS (*β* = 0.128; *p* = 0.002; Table 6), in regard of components of MetS, rs237025*WM is an independent risk factor for new-onset increased WC (*β* = 0.116; *p* = 0.001; Table 6), elevated blood pressure (*β* = 0.224; *p* < 0.001; Table 6), decreased HDL-c (*β* = 0.148; *p* < 0.001; Table 6), and Elevated FPG (*β* = 0.145; *p <* 0.001; Table 6). The interaction of rs237025*WM and MetS is independent from age, sex, and pharmaceutical treatments.

**TABLE 6:** Liner regression analysis of changes in diagnose of MetS and MetS components.

DV	IV	Standard β	*p*	Adjusted R^2^
MetS (alleviating = 0, maintaining = 1, new-onset = 2)				0.730
	rs237025*WM	0.128	0.002	
	Sex	−0.210	<0.001	
	Age	0.928	<0.001	
	AH	−0.159	<0.001	
	AD	0.064	<0.001	
	Statin	0.051	0.001	
Increased WC (alleviating = 0, maintaining = 1, new-onset = 2)				0.800
	rs237025*WM	0.116	0.001	
	Sex	−0.171	<0.001	
	Age	0.955	<0.001	
	AH	−0.073	<0.001	
	AD	−0.036	0.013	
Elevated BP (alleviating = 0, maintaining = 1, new-onset = 2)				0.891
	rs237025*WM	0.224	<0.001	
	Sex	−0.046	<0.001	
	Age	0.837	<0.001	
	AH	−0.108	<0.001	
Elevated TG (alleviating = 0, maintaining = 1, new-onset = 2)				0.632
	rs237025*WM	0.043	0.378	
	Sex	−0.095	<0.001	
	Age	0.850	<0.001	
	AH	−0.113	<0.001	
	Statin	0.135	<0.001	
Decreased HDL-c (alleviating = 0, maintaining = 1, new-onset = 2)				0.782
	rs237025*WM	0.148	<0.001	
	Sex	−0.107	<0.001	
	Age	0.814	<0.001	
Elevated FPG (alleviating = 0, maintaining = 1, new-onset = 2)				0.754
	rs237025*WM	0.145	<0.001	
	Sex	−0.090	<0.001	
	Age	0.790	<0.001	
	AH	−0.085	<0.001	
	AD	0.135	<0.001	

DV, dependent variables; IV, independent variables; MetS, metabolic syndrome; rs237025, AA/AG = 0, GG = 1; WM, weight loss = 0, weight gain = 1; AH, no antihypertensive = 0, antihypertensive = 1; AD, no antidiabetic = 0, antidiabetic = 1; Statin, no statin = 0, statin = 1; WC, waist circumference; BP, blood pressure; TG, triglyceride; HDL-c, High-density Lipoprotein-cholesterol; FPG, fasting plasma glucose.

To elucidate the interaction of rs237025 and weight management, we further conducted a cross-over analysis under the logistic regression model. The synergistic effect of rs237025 and weight management on WC is negative (*p* < 0.001; S = 0.564; Supplementary Table S6); the synergistic effect of rs237025 and weight management on TG is negative (*p* = 0.005; S = 0.639; Supplementary Table S6); the synergistic effect of rs237025 and weight management on HDL-c is negative (*p* < 0.001; S = 0.750; Supplementary Table S6); the synergistic effect of rs237025 and weight management on WC is negative (*p* < 0.001; S = 0.564; Supplementary Table S6).

## Discussion

This study has identified the *SUMO4* gene polymorphism rs237025 as an independent risk factor for MetS in the Shandong China population. The rs237025 polymorphism is positively related to risks of having increased WC, elevated TG, and elevated FPG. The follow-up survey has found rs237025 is positively related to the risk of decreased HDL-c in patients without pharmaceutical therapy. Cross-over analysis of the gene-environment has identified the interaction of rs237025 and weight management as a risk factor for MetS, increased WC, elevated blood pressure, decreased HDL-c, and increased FPG.

Kurt Bohren and colleagues found *SUMO4* gene rs237025 polymorphism related to T1D susceptibility (Bohren et al., 2004), and later on, Dehuang Guo and colleagues confirmed in their multiethnic study that the G allele is significantly increased in T1D patients (Guo et al., 2004). However, regarding the susceptibility of T1D in different ethics differs in multiple other studies, T1D susceptibility of rs237025 mutants was significantly increased in the east Asia population (Qu et al., 2005; Smyth et al., 2005), but not in Caucasians (Noso et al., 2005). Similar results were reported in T2D susceptibility (Shimada et al., 2009; Ji et al., 2011; Sozen et al., 2014), a meta-analysis has thoroughly reviewed published works, and recognized rs237025 polymorphism as a risk factor for diabetes in the Chinese population, but not in other ethnicities (Zhang et al., 2017). These studies suggest a race-depended rs237025 pathogenicity, in accordance with previous research on East Asian populations, our study finds that rs237025 GG genotype is abundant in people with increased FPG and that rs237025 under the recessive model is an independent risk factor for elevated FPG.

NF-κB binds to the SUMO4 promoter and activates its transcription, therefore SUMO4 act as a negative feedback regulator of NF-κB (Wang et al., 2009). On the other hand, SUMO4 combines and modifies IκBα, by which it inhibits NF-κB-dependent transcription of IL-12B, rs237025 is a nonsynonymous mutation which leads to a 5.5-times increase of NF-κB, and the expression of IL-12B increases by 2 times (Guo et al., 2004). NFκB is involved in the insulin-regulated liver glucose metabolism, high glucose-induced apoptosis, and is reportedly related to the insulin resistance of adipocytes (Heo et al., 2019; Tang et al., 2020). Therefore, the nonsynonymous mutation of SUMO4 may lead to an elevated expression of NF-κB, which causes insulin resistance and T2D. In addition, NF-κB is reported to be elevated in autoimmune disease. SUMO4 rs237025 relates to activation of immune cells and contributes to the autoimmune response, including T1D, autoimmune thyroid disease, and rheumatoid arthritis (Tsurumaru et al., 2006), however, susceptibility of rheumatoid arthritis or juvenile idiopathic arthritis was not associated with rs237025 polymorphism in the English Caucasian population (Gibbons et al., 2005). Increased peripheral insulin resistance and autoimmune response in rs237025 variants may partially explain the elevated FPG.

This study identified that rs237025 polymorphism is associated with increased WC and elevated TG. The lipid metabolism and the glucose metabolism are mutually regulated, and increased blood glucose could lead to splanchnic and peripheral lipid deposition which will further exacerbate insulin resistance and glucose intolerance. Systemic metabolic disorders of glucose and lipids are the pathogenic base of MetS. Accordingly, the cross-section study identified rs237025 as a risk factor for MetS.

The follow-up study shows that rs237025 is related to increased incidence of newly diagnosed low HDL-c, however, change in HDL-c as a continuous variable was not significantly related to rs237025 polymorphism. We believe that the HDL-c level in previously diagnosed low HDL-c patients is largely affected by pre-exposures, and a critical value for diagnosing low HDL-c would be more sensitive to notice. Therefore, the qualitative variables compare to the quantitative variables are more significant in this research. Unfortunately, the follow-up study failed to relate SNP rs237025 with MetS directly. MetS is a result of the accumulative effects of genetic predisposition and environment, and participants in this research were allowed to have pharmaceutical intervention in the 5-year period, therefore, the risk of rs237025 on MetS is largely affected by confounding variates. We studied the additive effect of gene polymorphism with weight management, and considered drug administration as a concomitant variable. The interaction of rs237025 and weight management is a risk factor for MetS and the components of MetS. SNP rs237025 is an inherited risk factor that is inevitably affected by the cumulative effect of time and environment, instead, weight management is predominately affecting the MetS related parameters in the 5-years survey. In other words, rs237025 was involved in the pathogenesis of MetS in an accessional manner during the 5-year period. This find is in accordance with the guideline recommendation that MetS patients should have a goal of 5% body weight loss. Furthermore, cross-over analysis indicates that the synergistic effect of rs237025 and weight management is negative, indicating that patients with rs237025 variants are prone to have decreased WC, decreased TG, and increased HDL-c when body weight is well-managed. In short, this study reassured weight management as the foundation of MetS intervention, and patients with rs237025 polymorphism are more likely to be benefited from weight reduction.

Unfortunately, there are some inherent limitations in this study. First of all, SNP rs237025 in the Increased WC group and the Elevated FPG does not meet the Hardy-Weinberg genetic equilibrium, which may be because of the sampling bias or limited sample scale. A conspicuous number of participants in the control group were dropped compared with the MetS group, which significantly reduces the statistical power of the research. On the other hand, laboratory indicators such as postprandial blood glucose, fasting insulin and C peptide, electrolytics, and hormone levels were not examined in this study, therefore insulin resistance and endocrine hormone disorder cannot be evaluated.

In conclusion, this study is the first study to identify *SUMO4* gene rs237025 polymorphism as an independent risk factor for MetS. The gene-environment interaction analysis has recognized the negative synergistic effect of rs237025 polymorphism and weight management, and highlighted the importance of weight management for MetS.

## Data Availability

ZW and MZ hold the access to the original data of this research and are responsible for the integrity of this work, the data is available for reasonable request.
