# Supplementary material for: SUMO4 Gene SNP rs237025 and the Synergistic Effect With Weight Management: A Study of Risk Factors and Interventions for MetS
Source: Front Genet. 2021 Dec 10;12:786393. doi: 10.3389/fgene.2021.786393 (PMC8709540; doi:10.3389/fgene.2021.786393)
Supplement: Supplementary file 1 [file DataSheet1.pdf]

Formula:

$$S = \frac{e^{\beta_a+\beta_b+\beta_{a*b}} - 1}{e^{\beta_a} + e^{\beta_b} - 2}$$

S stand for the synergistic index of cross-over analysis; β_a is the coefficient of rs237025 under logistic regression model, β_b is the coefficient of weight management under logistic regression model, β_{a*b} is the coefficient of the additive effect of rs237025 and weight management under logistic regression model

Supplementary Table 1 Hardy-Weinberg genetic equilibrium of rs237025

	AA		AG		GG		χ^2	*p*
	OF	EF	OF	EF	OF	EF		
Control (n=1047)	532	527.27	422	431.47	93	88.27	0.504	0.777
MetS (n=1008)	481	465.50	408	439.00	119	103.50	5.026	0.081
Total (n=2055)	1013	992.30	830	871.39	212	191.30	4.637	0.098
Normal WC (n=1136)	573	570.44	464	469.11	99	96.44	0.135	0.935
Increased WC (n=919)	440	422.34	366	401.32	113	95.34	7.119	0.028
Normal BP (n=919)	468	459.03	363	380.94	88	79.03	2.037	0.361
Elevated BP (n=1136)	545	533.51	467	489.99	124	112.51	2.501	0.286
Normal TG (n=1336)	674	666.31	539	554.38	123	115.31	1.028	0.598
Elevated TG (n=719)	339	326.48	291	316.04	89	76.48	4.512	0.105
Normal HDL-c (n=1692)	833	816.67	685	717.67	174	157.67	3.506	0.173
Decreased HDL-c (n=363)	180	175.64	145	153.73	38	33.64	1.170	0.557
Normal FPG (n=1534)	769	762.48	625	638.04	140	133.48	0.641	0.726
Elevated FPG (n=521)	244	230.45	205	232.11	72	58.45	7.107	0.029

OF, Observed Frequency; EF, Expected Frequency; MetS, Metabolic Syndrome; WC, Waist Circumference; BP, Blood Pressure; TG, Triglyceride; HDL-c, High-density Lipoprotein-cholesterol; FPG, Fasting Plasma Glucose

Supplementary Table 2 Characteristic of M55V genotypes under recessive model in the Control group and the MetS group

	Control			MetS		
	AA/AG (n=954)	GG (n=93)	p	AA/AG (n=889)	GG (n=119)	*p*
Height (cm)	162.08 ± 7.74	160.19 ± 6.64	0.024	162.59 ± 7.71	163.62 ± 8.17	0.174
Weight (kg)	58.76 ± 8.74	57.27 ± 8.02	0.114	72.43 ± 10.86	73.13 ± 11.67	0.508
WC (cm)	76.28 ± 6.76	76.42 ± 6.37	0.848	90.99 ± 8.42	92.71 ± 8.49	0.036
BMI (kg/m2)	22.35 ± 2.77	22.31 ± 2.70	0.893	27.37 ± 3.42	27.26 ± 3.43	0.735
SBP mmHg	119.74 ± 9.73	121.46 ± 12.96	0.116	149.69 ± 19.73	148.01 ± 19.88	0.385
DBP mmHg	75.10 ± 6.74	76.11 ± 8.68	0.180	90.99 ± 10.85	89.89 ± 10.06	0.293
TC (mmol/L)	4.16 ± 0.57	4.20 ± 0.61	0.500	4.70 ± 1.19	4.80 ± 1.18	0.377
TG (mmol/L)	0.96 ± 0.45	1.07 ± 0.59	0.037	2.35 ± 1.64	2.46 ± 1.74	0.516
HDL-c (mmol/L)	1.58 ± 0.34	1.61 ± 0.35	0.451	1.49 ± 0.53	1.48 ± 0.51	0.808
LDL-C (mmol/L)	2.58 ± 0.54	2.59 ± 0.56	0.810	3.20 ± 0.82	3.30 ± 0.83	0.207
FPG (mmol/L)	4.62 ± 0.65	4.70 ± 0.63	0.215	5.94 ± 2.10	6.44 ± 2.34	0.017

MetS, Metabolic Syndrome; BMI, Body Mass Index; WC, Waist Circumference; SBP, Systolic Blood Pressure; DBP, Diastolic Blood Pressure; TG, Triglyceride; TC, Total Cholesterol; HDL-c, High-density Lipoprotein-cholesterol; LDL-c, Low-density Lipoprotein-cholesterol; FPG, Fasting Plasma Glucose

Supplementary Table 3 χ^2 analysis of newly diagnosed decreased HDL-c without drug administration

	Newly diagnosed decreased HDL-c		Others		χ^2	*p*
	Counts	Frequency	Counts	Frequency		
AA/AG	113	26.40%	315	73.60%	5.154	0.023
GG	26	40.00%	39	60.00%		

HDL-c, High-density Lipoprotein-cholesterol

Supplementary Table 4 Logistic regression of newly diagnosed decreased HDL-c without pharmaceutical therapy

	p	OR	OR 95% C.I.	
			lower limit	upper limit
rs237025 (AA/AG=0, GG=1)	0.016	2.058	1.143	3.707
Sex (Female=0, Male=1)	<0.001	0.291	0.176	0.479
Height (cm)	<0.001	0.982	0.975	0.988
TC (mmol/L)	<0.001	1.682	1.340	2.112

TC, Total Cholesterol; OR, Odds Ratio

Supplementary Table 5 Logistic regression analysis of weight management

	p	OR	OR 95% C.I.	
			lower limit	upper limit
Salt restriction (no=0, yes=1)	<0.001	2.859	1.978	4.133
Exercises (no=0, yes=1)	<0.001	2.241	1.510	3.324
Diet (no=0, yes=1)	<0.001	2.817	1.732	4.582
Antihypertensives (no=0, yes=1)	<0.001	4.034	2.842	5.726
Antidiabetics (no=0, yes=1)	0.024	1.861	1.083	3.199

Supplementary Table 6 Cross-over analysis of M55V and WM under logistic regression model

DV	IV	β	*p*	S
WC (decreased=0, increased=1)				0.564
	rs237025	-1.508	<0.001	
	WM	-3.869	<0.001	
	rs237025*WM	0.720	<0.001	
SBP (decreased=0, increased=1)				0.685
	rs237025	-0.584	0.103	
	WM	-0.872	0.006	
	rs237025*WM	0.247	0.109	
DBP (decreased=0, increased=1)				0.824
	rs237025	-0.112	0.755	
	WM	-1.194	<0.001	
	rs237025*WM	0.223	0.147	
TG (decreased=0, increased=1)				0.639
	rs237025	-0.770	0.034	
	WM	-1.536	<0.001	
	rs237025*WM	0.440	0.005	
HDL-c (decreased=0, increased=1)				0.750
	rs237025	1.430	<0.001	
	WM	0.896	0.009	
	rs237025*WM	-0.828	<0.001	
FPG (decreased=0, increased=1)				0.663
	rs237025	-0.078	0.826	
	WM	-0.027	0.931	
	rs237025*WM	0.035	0.819	

DV, Dependent Variables; IV, Independent Variables; rs237025, AA/AG=0, GG=1; WM, weight loss=0, weight gain=1; WC, Waist Circumference; SBP, Systolic Blood Pressure; DBP, Diastolic Blood Pressure, TG, Triglyceride; HDL-c, High-density Lipoprotein-cholesterol; FPG, Fasting Plasma Glucose

Supplementary Table 7 Demographic information of participants in cross-section survey and follow-up survey

	Age (years old)			Total
	<40	40-60	≥60	
Cross-section survey	233	1397	425	2055
Follow-up survey	43	646	451	1140
